# Characteristics of moderate-to-severe chronic pain and its association with daily functional impairment among older outpatients in Vietnam

**DOI:** 10.1371/journal.pone.0335234

**Published:** 2025-11-10

**Authors:** Thanh Xuan Nguyen, Thu Thi Hoai Nguyen, Huong Thi Thu Nguyen, Tam Ngoc Nguyen, Hong Trang Nguyen, Linh Huyen Vu Ha, Hoa Trung Dinh, Luc Viet Tran, Anh Trung Nguyen, Hoa Lan Nguyen, Robert Joel Goldberg, Janani Thillainadesan, Vasikaran Naganathan, Huyen Thi Thanh Vu

**Affiliations:** 1 Hanoi Medical University, Hanoi, Vietnam; 2 National Geriatric Hospital, Hanoi, Vietnam; 3 Phenikaa university, Hanoi, Vietnam; 4 National Hospital of Endocrinology, Hanoi, Vietnam; 5 Department of Population and Quantitative Health Sciences, University of Massachusetts Medical School, Worcester, Massachusetts, United States of America; 6 Department of Geriatric Medicine and Centre for Education and Research on Ageing (CERA), Concord Hospital, Sydney, Australia; 7 Faculty of Medicine and Health, University of Sydney, Sydney, Australia; Bach Mai Hospital, VIET NAM

## Abstract

**Objective(s):**

To describe the characteristics of moderate-to-severe chronic pain, and its association with daily functional decline among older individuals attending a large outpatient geriatric clinic in Hanoi, Vietnam.

**Methods:**

A cross-sectional study was undertaken between November, 2019 and March, 2020. In-person structured interviews were conducted in 518 patients 60 years and older with chronic pain. Patient’s self-reported pain was categorised into two levels of mild (0–3), moderate to severe (4–10) pain based on a 0–10 scale.

**Results:**

The median age of the study sample was 69 years, women accounted for 74.1%.

The knee-foot-leg region was the most common site of chronic pain in both groups (39.5% in mild pain vs. 45.1% in moderate-to-severe pain. Participants experiencing moderate-to-severe pain were more likely to describe their pain as dull (65.5%) or burning (11.3%) and to report pain occurring independently of specific activities (48.7%), compared to those with mild pain (53.9%, 1.2%, and 32.4%, respectively). In a multivariable adjusted model, dependency in activities of daily living (OR: 3.73, 95% CI: 1.82–7.68) and dependency in instrumental activities of daily living (OR: 9.41, 95% CI: 3.94–22.5) were significantly associated with moderate-to-severe pain.

**Conclusions:**

Moderate to severe chronic pain was reported in a high proportion of older Vietnamese outpatients and was associated with impaired daily functional impairment.

## Introduction

Chronic pain is pain that lasts for three or more months or longer than expected [1]. While pain is not an inherent aspect of aging, its prevalence increases with advancing age [2]. Worldwide, approximately one-third of adults report chronic pain with this rate being higher among both community-dwelling and institutionalized older adults [3–5]. Chronic unrelieved pain significantly reduces one’s health condition and causes chronic disability [6].

The prevalence and characteristics of chronic pain in older adults vary across studies due to differences in demographics, healthcare access, and disease burden [7]. Common causes include musculoskeletal disorders, osteoarthritis, neuropathic pain, and inflammatory conditions. Age-related physiological changes, such as reduced pain tolerance and neurodegeneration, further complicate pain assessment and management [8]. The commonest sites of pain are the back, leg (knee or hip) [9]. Pain severity varies significantly among older adults and plays a crucial role in determining its impact on daily life [10,11]. Pain intensity is commonly classified as mild, moderate, or severe using validated tools [12]. The prevalence of moderate to severe pain among older adults with chronic pain is notably high, ranging from 25% to 50% across different studies [13]. Older adults with moderate-to-severe chronic pain experience greater disability, lower physical performance, and increased reliance on assistive devices compared to those with mild pain [14]. Furthermore, evidence suggests that higher pain intensity is associated with increased healthcare utilization, greater medication burden, and poorer treatment outcomes [15,16].

Vietnam is undergoing a rapid demographic transition, with a growing proportion of older adults, making chronic pain a pressing public health concern. Factors such as rising life expectancy, the increasing burden of non-communicable diseases, and socioeconomic transitions affecting healthcare access contribute to the escalating prevalence of chronic pain in this population [17]. Despite this trend, the severity, characteristics, and functional impact of chronic pain, particularly moderate-to-severe pain, remain underexplored among older adults in Vietnam. A deeper understanding of the distribution of pain severity and its differential effects on daily functioning is essential for optimizing pain management strategies in this demographic. This study aims to describe the characteristics of moderate-to-severe chronic pain, and its association with decline of functionamong older individuals attending a large outpatient geriatric clinic in Hanoi, Vietnam. By comparing individuals with mild chronic pain to those with moderate-to-severe pain, this research seeks to elucidate how pain severity differentially affects functional status. Findings from this study will provide critical insights to inform tailored pain management approaches, ultimately improving clinical outcomes and enhancing the quality of life for older adults in Vietnam.

## Methods

### Study setting

A cross-sectional study was conducted in the outpatient department of a leading specialized geriatric hospital in Vietnam, from November 2019 to March 30, 2020. As a leading national referral center for geriatric care, the hospital provides both outpatient and inpatient services, offering comprehensive medical care through a multidisciplinary approach for older adults. The outpatient clinic primarily serves individuals aged 60 years and older, in accordance with the definition of older adults in Vietnam.

### Study participants

Potential study participants were recruited from the outpatient department. The participants in the study included persons who were 60 years of age or older and had chronic pain (pain lasting ≥3 months) using the International Association for the Study of Pain (IASP) classification [18]. We excluded individuals with severe medical conditions (defined as requiring intensive care), mental disorders, or cognitive impairment, as well as those who declined to participate in the study.

### Sample size

The minimum required sample size was estimated using the formula for proportion estimation, assuming the expected proportion of patients with moderate to severe chronic pain among older patients of 50% to maximize the required sample size, with a 95% confidence level (Z = 1.96) and a margin of error of 5% (d = 0.05). This calculation yielded a minimum sample size of 384 participants. However, to enhance the robustness and generalizability of the findings, we included all eligible patients who met the inclusion criteria during the study period, resulting in a final sample size of 518.

### Data collection

Prior to recruiting participants, five nursing students completed training in screening and data collection approaches using the specific measures to be employed in the present study. Participants received a complete explanation of the purpose, risks, and procedures of the study and signed a written informed consent.

### Study measures and data collection instruments

Eligible and consenting study subjects were interviewed in-person in the outpatient department and additional information was obtained from a review of their hospital medical records. The interviews obtained self-reported information on demographic characteristics, pain characteristics, daily functional status, and other relevant health indicators. Clinical data, including comorbidities, medication use were extracted from participants’ medical records.

#### Sociodemographic, lifestyle characteristics.

Information on the patient’s age, sex, marital status, living status, monthly income, and lifestyle characteristics, including smoking and alcohol use, was collected from the in-person interview. Smoking status was categorized as either current smoker, former smoker, or never having smoked. Alcohol use was categorized as currently drinking alcohol or not.

Low-income status was defined in accordance with Vietnam Government Decree No. 30/2025/NĐ-CP, which identifies a low-income worker in urban areas as one with a monthly per-person income 117.7 USD or less.

#### Comorbidity.

Comorbidities were assessed by physicians using the Charlson Comorbidity Index (CCI) [19], which comprises 19 medical conditions. Each condition was assigned a weighted score ranging from 1 to 6, based on its estimated impact on 1-year mortality risk. The total CCI score serves as an indicator of overall disease burden, with higher scores reflecting greater comorbidity severity.

#### Daily functioning.

Daily functioning was evaluated using both basic and instrumental activities of daily living. Basic activities of daily living (ADLs) were measured with the Katz Index of Independence in ADLs, which assesses essential self-care tasks, including bathing, dressing, toileting, transferring, continence, and feeding. Instrumental activities of daily living (IADLs) were also assessed to evaluate an individual’s ability to perform more complex tasks necessary for independent living, such as meal preparation, housekeeping, medication management, and financial handling [20].

Daily functional impairment was defined according to standardized scoring criteria. The Katz ADL Index ranges from 0 to 6, with scores below 6 indicating impairment in basic daily activities. The IADL scale ranges from 0 to 8 for women and 0 to 5 for men, with scores below these thresholds considered indicative of impairment in instrumental daily functions. Both tools are routinely used in clinical practice and have been widely applied in previous research on older adults in Vietnam [21,22]

#### Depression.

Participants were directly interviewed using the 15-item Geriatric Depression Scale Vietnamese version to screen for depression. The total scores range from 0 to 15, with scores of 0-5 considered normal, and 6-15 indicating having depressive symptoms [23]

#### Social isolation.

The Lubben Social Network Scale was used to assess participant’s social isolation. Total scores range from 0-30 with higher scores indicating more social engagement. A person at high risk for social isolation was defined as a less than 20 score in the Lubben Social Network Scale [24].

#### Poor sleep.

Poor sleep was defined based on the Pittsburgh Sleep Quality Index (PSQI), a validated measure consisting of 19 items assessing various aspects of sleep quality. The total score ranges from 0 to 21, with a score greater than five indicating poor sleep [25]

#### Chronic pain assessment.

Pain lasting ≥3 months was defined as chronic pain following the IASP classification [18] chronic pain was measured by two questions: (1) “Do you have pain?”; (2) “If yes, how long have you had this pain?”.

Pain intensity was evaluated using the Numerical Rating Scale (NRS), an 11-point scale ranging from 0 (indicating no pain) to 10 (representing the worst pain imaginable) [12]. Participants were instructed to report their average pain intensity over the past week by selecting a score between 0 and 10. Based on the reported scores, pain severity was classified into three categories: mild (1–3), moderate (4–6), and severe (7–10).

Pain location was determined by asking study participants to look at a picture and point to their pain sites, which were classified as either 1, 2, or 3 or more locations.

If pain was present at more than one site, patients were asked about the most painful location. Study patients were asked about their daily pain frequency (once, twice, three or more times), pain characteristics (burning, sharp, dull), when they got pain (any time and not related to activities, during daytime with increased movement and activity, sleeping at night, during daytime with resting), factors associated with relieving and aggravating their pain, their use of pain medication (yes or no). Their level of satisfaction with current pain treatment was assessed with the question “Are you satisfied with your current pain treatment?” (yes/no)

### Data analysis

Categorical variables are presented as percentages. Continuous variables are presented as means ± standard deviation or as medians (interquartile range)

Comparisons between participants with mild pain and those experiencing moderate to severe pain were conducted using the Chi-squared test for categorical variables and either ANOVA or the Kruskal-Wallis test for continuous variables. Unadjusted and adjusted logistic regression models with functional dependency (ADL and IADL) as the dependent variable were used to investigate the relationship between moderate to severe pain and functional dependency. Variables incorporated into the multivariable models were selected based on their clinical relevance and supporting evidence from the literature. Model 1 was unadjusted regression between moderate to severe pain with functional dependency. Age and gender were included in the adjusted model 2. Model 3 were including age, gender and pain characteristics (total pain location, using analgesic and satisfaction with current pain treatment). The final model included these factors and other confounding factors (having depression symptom, poor sleep and social isolation). The results are presented as multivariable adjusted odds ratios and 95%CI. Data analyses were conducted in SPSS version 23.0.

### Ethical consideration

The study protocol was approval by the Ethic Committee of National Geriatric Hospital, Hanoi, Vietnam with the number 635/IRB-NGH on 11^th^ October 2019. The participants have given written informed consent.

## Results

The median age of the 518 study participants was 69 years, 74.1% were women, the majority were married, and nearly one-half lived in rural areas (Table 1). Approximately 47% of participants were categorized as having mild chronic pain, while 53.1% were classified in the moderate-to-severe pain group at the time of clinical assessment. The median age was comparable between the two groups (68 years in the mild pain group vs. 70.8 years in the moderate-to-severe pain group, p = 0.35). A significantly higher proportion of female participants was observed in the mild pain group (80.2%) compared to the moderate-to-severe pain group (68.7%) (p = 0.003). The Charlson comorbidity score was slightly higher in the moderate-to-severe pain group (mean = 1.2, SD = 1.4) compared to the mild pain group (mean = 0.9, SD = 1.3), the difference did not reach statistical significance (p = 0.06).

**Table 1:** Baseline characteristics of outpatients according to severity of chronic pain.

Characteristic	Chronic Pain		
	Mild (n = 243, 46.9%) n (%)	Moderate and severe (n = 275, 53.1%) n (%)	p Value
Age, median (IQR) years	68 (11.0)	70.8 (8.5)	0.35
Female	195 (80.2)	189 (68.7)	0.003
Married	183 (75.3)	219 (79.6)	0.24
Rural residence	133 (43.8)	171 (62.2)	0.09
Low income	13 (5.4)	6 (2.2)	0.2
Drink alcohol (current)	22 (9.4)	25 (9.1)	0.43
Smoking (current)	6 (2.6)	12 (4.4)	0.14
Charlson comorbidity score Mean (SD)	0.9 (1.3)	1.2 (1.4)	0.06

SD: standard deviation; IQR: interquartile range, t-test, Chi-square test

Table 2 presented the pain characteristics of participants stratified by chronic pain severity. Among the 518 participants, the most commonly reported pain locations in both groups were the knee-foot-leg (39.5% in mild pain vs. 45.1% in moderate-to-severe pain) and head-face-neck (29.2% vs. 31.6). A majority of participants experienced pain once daily (63.0% in mild pain vs. 60.4% in moderate-to-severe pain), while a smaller proportion reported pain occurring three or more times per day (10.7% vs. 9.8%). Dull pain was the most frequently reported type, particularly among those with moderate-to-severe pain (65.5% vs. 53.9%). Burning pain was reported by a greater proportion of participants in the moderate-to-severe pain group (11.3%) compared to the mild pain group (1.2%). Participants with moderate-to-severe pain were less likely to experience pain during the daytime with increased movement and activity (37.5%) compared to those with mild pain (48.6%). With regard to factors relieving pain, resting was the most commonly reported alleviating factor in both groups (84.0% vs. 76.0%), followed by painkillers, which were used more frequently by those in the moderate-to-severe pain group (43.3% vs. 31.7%). Other relieving factors such as warm compress (10.9% vs. 3.7%) and changing body position (8.7% vs. 3.3%) were reported more frequently in the moderate-to-severe pain group.

**Table 2:** Pain characteristics in outpatients according to severity of chronic pain (n = 518).

Pain characteristic	Mild (n = 243) n (%)	Moderate and severe (n = 275) n (%)
**Location**		
Knee-Foot-Leg	96 (39.5)	124 (45.1)
Head-Face-Neck	71 (29.2)	87 (31.6)
Back-Hip	57 (23.5)	64 (23.3)
Shoulder-Arm-Elbow-Hand	56 (23.0)	73 (26.5)
Other	51 (21.0)	32 (11.6)
Stomach-Abdomen	31 (12.8)	33 (12.0)
**Pain frequency (daily)**		
Once	153 (63.0)	166 (60.4)
Twice	64 (26.3)	82 (29.8)
Three or more times	26 (10.7)	27 (9.8)
**Characteristics**		
Burning	3 (1.2)	31 (11.3)
Sharp	50 (20.6)	59 (21.5)
Dull	131 (53.9)	180 (65.5)
**When do participants get pain**		
Any time and not related to activities	112 (46.1)	138 (50.2)
During daytime with increased movement an activity	118 (48.6)	103 (37.5)
Sleeping at night	66 (27.2)	28 (10.2)
During daytime with resting	47 (19.3)	38 (13.8)
**Factors relieving pain**		
Resting	204 (84.0)	209 (76.0)
Pain killers	77 (31.7)	119 (43.3)
Sleeping	35 (14.4)	39 (14.2)
Changing body position	8 (3.3)	24 (8.7)
Warm compress	9 (3.7)	30 (10.9)
Stimulants (i.e., alcohol and coffee)	0 (0.0)	1 (0.4)
Consuming a full meal	0 (0.0)	4 (1.5)
**Factors aggravating pain**		
Walking	115 (47.3)	143 (52.0)
Changing body position	59 (24.3)	120 (43.6)
Insomnia	64 (26.3)	70 (25.5)
Change in weather	157 (64.6)	158 (57.5)
Stress	59 (24.3)	56 (20.4)
Eating	27 (11.1)	17 (6.2)
Stimulants	3 (1.2)	8 (2.9)

Table 3 presents the associations between moderate-to-severe pain and functional dependency in ADL and IADL. In the unadjusted model, individuals with moderate-to-severe pain had significantly higher odds of ADL dependency (OR: 1.83, 95% CI: 1.24–2.72) and IADL dependency (OR: 2.72, 95% CI: 1.81–4.07). These associations persisted after adjusting for age and gender (Model 2) and were further strengthened when accounting for total pain location, analgesic use, and satisfaction with pain treatment (Model 3). In the fully adjusted model (Model 4), which additionally included depression symptoms, poor sleep, and social isolation, the associations remained robust, with ORs of 3.73 (95% CI: 1.82–7.68) for ADL dependency and 9.41 (95% CI: 3.94–22.5) for IADL dependency.

**Table 3:** Results from logistic regression models examining association between moderate to severe pain and functional dependency (n = 518).

	Odds Ratios (CI 95%) for ADL dependent	Odds Ratios (CI 95%) for IADL dependent
**Unadjusted model**		
**Model 1**	1.83 (1.24-2.72)	2.72 (1.81-4.07)
**Adjusted models**		
**Model 2**	1.67 (1.09-2.55)	2.59 (1.66-4.03)
**Model 3**	3.93 (1.98-7.79)	9.3 (4.09-21.3)
**Model 4**	3.73 (1.82-7.68)	9.41 (3.94-22.5)

**Model 1:** Unadjusted

**Model 2:** Adjusted for age and gender

**Model 3:** Adjusted for age, gender, total pain location, using analgesis**,** satisfaction level with current pain treatment

**Model 4:** Adjusted for age, gender, total pain location, using analgesis**,** Satisfaction level with current pain treatment, having depression symptom, poor sleep and social isolation

## Discussion

The results of this cross-sectional study among more than 500 older outpatients with chronic pain treated at a large hospital in Hanoi showed that more than one-half reported moderate to severe pain. The knee-foot-leg was the most common site of chronic pain, and moderate to severe chronic pain was associated with daily functional impairment.

The prevalence of moderate to severe pain among older adults varies across studies, with estimates ranging from 25% in community-dwelling individuals, over 50% in nursing home residents and over 50% in hospital populations [26–28]. These discrepancies may result from differences in study populations, healthcare settings, and definitions of chronic pain [29].

The notably high prevalence of moderate to severe pain in our study could be partly due to underdiagnosis and undertreatment in older patients. To address this issue, it is essential to enhance healthcare professionals’ competencies in pain assessment and management and to implement systematic, comprehensive pain evaluation strategies. Such efforts are crucial to improving the quality of life and overall care for older adults in Vietnam.

In this study, the knee, foot, and leg were the most frequently reported pain sites across both mild and moderate-to-severe pain groups. This result is consistent with previous findings indicating that chronic musculoskeletal pain is among the predominant contributors to chronic pain in older adults [9]. Notably, participants experiencing moderate-to-severe pain were more likely to describe their pain as burning or dull and to report experiencing pain independent of specific activities. These characteristics suggest an increased likelihood of neuropathic or centrally mediated pain mechanisms within this subgroup. Furthermore, differences in pain management strategies were observed; participants with more severe pain demonstrated a greater reliance on pharmacological interventions. These findings emphasize the need for a more comprehensive and multimodal pain management approach for older adults with moderate to severe pain. Given their greater reliance on pharmacological interventions, optimizing medication use—while minimizing potential side effects—should be a priority. Non-pharmacological strategies, including physical therapy, cognitive-behavioral therapy, and tailored exercise programs, should also be integrated to address both nociceptive and neuropathic components of pain [30].

Activities of Daily Living play a crucial role in maintaining independence, quality of life, and reducing the risk of health decline in older adults, making them a key target in geriatric treatments and interventions [31]. In our study, we utilized the Visual Analog Scale to assess pain intensity, as it was the primary tool used in clinical practice at the time for evaluating pain severity in Vietnam, making it a practical and widely accepted choice for assessing moderate-to-severe chronic pain. Our findings indicated that the presence of moderate to severe chronic pain was related to daily functional impairment even after adjustment for several potentially confounding variables. This aligns with prior research demonstrating a relationship between chronic pain severity and functional impairment. In a cross-sectional analysis of middle-aged and older persons, approximately one-fifth of men and one-quarter of women with moderate to severe pain-impaired regular activities [32]. The results of a two-year longitudinal study of older adults showed that moderate/ severe pain was related to a significantly increased risk of disability [33]. The mechanisms underlying the relationship between chronic pain and functional decline are multifaceted. Persistent and inadequately managed pain contributes to physical deconditioning, reduced mobility, and fear of movement, further exacerbating disability [8]. Additionally, chronic pain has been implicated in the development of frailty and sarcopenia, both of which are associated with an increased risk of loss of independence [34,35]. The interplay between intrinsic factors (e.g., pain perception, psychological distress, comorbidities) and extrinsic factors (e.g., inadequate pain management, environmental barriers) likely contributes to the progressive burden of pain on functional capacity. From a clinical perspective, these findings underscore the necessity of a proactive, multidimensional approach to pain management in older adults. Interdisciplinary interventions, including physical therapy, occupational therapy, and geriatric rehabilitation, may help mitigate the impact of chronic pain on daily activities. Furthermore, routine pain assessments should be integrated into geriatric care to identify individuals at risk of functional decline, facilitating early intervention and targeted management strategies. Future longitudinal studies are needed to elucidate the causal pathways linking pain severity to functional impairment and to develop evidence-based interventions that not only alleviate pain but also preserve functional independence. Understanding the long-term impact of pain on disability trajectories will be essential for optimizing geriatric pain management strategies.

To our knowledge this is the first study to determine the characteristics of pain and evaluate the factors associated with moderate to severe chronic pain in older outpatients in Vietnam. There are some limitations of the study to consider. This investigation was cross-sectional and study participants were recruited from a tertiary care medical center so the findings may not be representative of older individuals living in the community. This study defines moderate-to-severe chronic pain using the Visual Analog Scale, which measures pain intensity but does not account for functional impairment. While Visual Analog Scale is widely used and was the standard clinical tool at the time of the study, this approach may limit comparability with other frameworks that incorporate disability measures. Another limitation is that alcohol use was only classified as current drinking or not, which may have limited our ability to fully adjust for its effects.

Our findings have implications for clinical practice and service delivery in Vietnam, particularly in the development of primary care and ambulatory hospital services. These services should incorporate evidence-based approaches to the screening, recognition, and treatment of chronic pain in older adults.

## Conclusions

Moderate to severe chronic pain was present in over 50% of older Vietnamese outpatients and was associated with impaired physical functioning as compared to less intense chronic pain. The knee, foot, and leg were the most common sites of chronic pain. Moderate to severe chronic pain was associated with impairment in daily functioning. Given the structure of Vietnam’s healthcare system, particularly within primary care and ambulatory services, a more comprehensive and integrated approach to chronic pain management in older adults is warranted to enhance clinical outcomes and maintain functional independence.
